# 1150. Resistance Analysis in the COMET-TAIL Study: Participants with Mild-to-Moderate COVID-19 Treated with Intramuscular or Intravenous Sotrovimab

**DOI:** 10.1093/ofid/ofac492.988

**Published:** 2022-12-15

**Authors:** Maria L Agostini, Gretja Schnell, Julia di Iulio, Anita Kohli, Adrienne E Shapiro, Elias H Sarkis, Dave Inman, Amanda Peppercorn, Andrew Skingsley, Leah A Gaffney, Melissa Aldinger, Christy M Hebner, Andrea L Cathcart

**Affiliations:** Vir Biotechnology, Inc., San Francisco, California; Vir Biotechnology, Inc., San Francisco, California; Vir Biotechnology, Inc., San Francisco, California; Arizona Liver Health, Tucson, Arizona; University of Washington and Fred Hutchinson Cancer Center, Seattle, Washington; Sarkis Clinical Trials, Gainesville, Florida; GlaxoSmithKline, Middlesex, England, United Kingdom; GlaxoSmithKline, Middlesex, England, United Kingdom; GlaxoSmithKline, Middlesex, England, United Kingdom; Vir Biotechnology, Inc., San Francisco, California; Vir Biotechnology, Inc., San Francisco, California; Vir Biotechnology, Inc., San Francisco, California; Vir Biotechnology, Inc., San Francisco, California

## Abstract

**Background:**

Sotrovimab (VIR-7831) is an engineered human monoclonal antibody targeting a conserved region of the SARS-CoV-2 spike protein; it has been shown to have a favorable safety profile and be effective for early treatment of high-risk COVID-19 patients. The COMET-TAIL phase 3 study evaluated sotrovimab administered intravenously (IV) or intramuscularly (IM) for the treatment of participants with mild to moderate COVID-19 who are at high risk of disease progression.

**Methods:**

Between June to August 2021, 973 participants were randomized and received sotrovimab by 500 mg IV infusion or by 500 or 250 mg IM injection. Deep sequencing of the spike gene was performed on nasopharyngeal samples. Baseline (BL; Day 1 or Day 3), post-BL (Day 5 or later), treatment-emergent (TE) substitutions at sotrovimab epitope positions, and presence of variants of concern/interest (VOC/VOI), were evaluated at a ≥5% allelic frequency. Phenotypic analyses were conducted using a pseudotyped virus assay.

**Results:**

Sequences were available from 764 participants (500 mg IV: 314/393; 500 mg IM: 302/387; 250 mg IM: 148/193). Consistent with VOC circulation during enrollment, the Delta variant was detected in 88.2% (674/764) of participants. Alpha and Mu variants were also seen at >2% prevalence. Of the 764 participants, 26 met the primary endpoint for clinical progression to hospitalization >24 hours or death due to any cause through day 29 and were infected with Delta (500 mg IV: 4; 500 mg IM: 9; 250 mg IM: 11), Alpha (500 mg IM: 1), or Mu (500 mg IV: 1) variants. Substitutions at sotrovimab epitope positions were similar across arms and were detected in 82/764 (10.7%) participants at any visit (500 mg IV: 42/314; 500 mg IM: 27/302; 250 mg IM: 13/148). Of these, 2 participants experienced clinical progression: 1 participant infected with the Mu variant (500 mg IV) carried the characteristic R346K substitution at BL; 1 participant infected with the Delta variant (500 mg IM) had P337L and E340K substitutions detected at Day 3 and P337L was enriched at Day 8. The predominant TE epitope substitutions included P337L and E340A/K/V, which confer reduced susceptibility to sotrovimab *in vitro*.

**Conclusion:**

Overall, TE epitope substitutions were not associated with clinical progression.

**Funding:**

Vir & GSK (NCT04913675)

**Disclosures:**

**Maria L. Agostini, PhD**, Vir Biotechnology, Inc: Employee|Vir Biotechnology, Inc: Stocks/Bonds **Gretja Schnell, PhD**, Vir Biotechnology: Employee|Vir Biotechnology: Stocks/Bonds **Julia di Iulio, PharmD, PhD, MBA**, Vir Biotechnology: Employee|Vir Biotechnology: Stocks/Bonds **Anita Kohli, MD**, GlaxoSmithKline: Third party funding to Vir|Vir Biotechnology Inc: Support as a trial site paid to my institution and non-financial support for medical writing support **Adrienne E. Shapiro, MD, PhD**, Vir Biotechnology: Support as a trial site paid to my institution and non-financial support for medical writing GlaxoSmithKline third party funding to Vir support **Elias H. Sarkis, MD**, Abbvie: Grant/Research Support|Abbvie: Speakers Bureau|Eisai: Grant/Research Support|GlaxoSmithKline: Third party funding to Vir|Ironshore: Grant/Research Support|Janssen: Speakers Bureau|Lilly: Grant/Research Support|Otsuka: Grant/Research Support|Teva: Speakers Bureau|Vir Biotechnology Inc: Support as a trial site paid to my institution and non-financial support for medical writing support **Dave Inman, MSc**, GlaxoSmithKline: Employee|GlaxoSmithKline: Stocks/Bonds **Amanda Peppercorn, MD**, GlaxoSmithKline: Employee|GlaxoSmithKline: Stocks/Bonds **Andrew Skingsley, MD**, GlaxoSmithKline: Employee|GlaxoSmithKline: Stocks/Bonds **Leah A. Gaffney, PhD**, Vir Biotechnology: Employee|Vir Biotechnology: Stocks/Bonds **Melissa Aldinger, PharmD**, Vir Biotechnology: Employee|Vir Biotechnology: Stocks/Bonds **Christy M. Hebner, PhD**, Vir Biotechnology: Employee|Vir Biotechnology: Stocks/Bonds **Andrea L. Cathcart, PhD**, Vir Biotechnology: Employee|Vir Biotechnology: Stocks/Bonds.

